# Assessing the effects of the percentage of chronic disease in households on health payment-induced poverty in Shaanxi Province, China

**DOI:** 10.1186/s12913-018-3698-1

**Published:** 2018-11-20

**Authors:** Xin Lan, Zhongliang Zhou, Yafei Si, Chi Shen, Xiaojing Fan, Gang Chen, Dantong Zhao, Xi Chen

**Affiliations:** 1 School of Public Health, Xi’an Jiaotong University Health Science Center, No. 76 Yanta West Road, Xi’an, 710061 Shaanxi China; 2 School of Public Policy and Administration, Xi’an Jiaotong University, No. 28 Xianning West Road, Xi’an, 710049 Shaanxi China; 3 Center for Health Economics (CHE), Monash University, Melbourne, Australia; 4 Department of Health Policy and Management and Department of Economics, Yale University, 60 College Street Suite 301, New Haven, CT 06510 USA

**Keywords:** China, Chronic diseases, Health payment-induced poverty, Logistic model, NHSS

## Abstract

**Background:**

Chronic disease has become one of the leading causes of poverty in China, which posed heavy economic burden on individuals, households and society, and accounts for an estimated 80% of deaths and 70% of disability-adjusted life-years lost now in China. This study aims to assess the effect of chronic diseases on health payment-induced poverty in Shaanxi Province, China.

**Methods:**

The data was from the 5th National Health Survey of Shaanxi Province, which was part of China’s National Health Service Survey (NHSS) conducted in 2013. Totally, 20,700 households were selected for analysis. We used poverty headcount, poverty gap and mean positive poverty gap to assess the incidence, depth and intensity of poverty before and after health payment, respectively. Logistic regression models were further undertaken to evaluate the influence of percentage of chronic patients in households on the health payment-induced poverty with the control of other covariates.

**Results:**

In rural areas, the incidence of poverty increased 31.90% before and after health payment in the household group when the percentage of chronic patients in the households was 0, and the poverty gap rose from 932.77 CNY to 1253.85 CNY (50.56% increased). In the group when the percentage of chronic patients in the households was 1–40% and 41–50%, the poverty gap increased 76.78 and 89.29%, respectively. In the group when the percentage of chronic patients in the households was 51~ 100%, the increase of poverty headcount and poverty gap was 49.89 and 46.24%. In the logistic model, we found that the proportion of chronic patients in the households was closely related with the health payment-induced poverty. The percentage of chronic disease in the households increased by 1 %, the incidence of poverty increased by 1.01 times. On the other hand, the male household head and the household’s head with higher educational lever were seen as protective factors for impoverishment.

**Conclusions:**

With the percentage of chronic patients in the households growing, the health payment-induced poverty increases sharply. Furthermore, the households members with more chronic diseases in rural areas were more likely to suffer poverty than those in urban areas. Our analysis emphasizes the need to protect households from the impoverishment of chronic diseases, and our findings will provide suggestions for further healthcare reforms in China and guidance for vulnerable groups.

## Background

China has been undergoing an epidemiological transition shifting from the infectious to the chronic diseases in the past three decades, which is much shorter than many other countries [1]. Chronic disease, defined as the diseases that affect people in a longer period in the future [2], mainly including cardiovascular diseases, diabetes, some cancers and so on [3], accounting for a majority of deaths and are responsible for a notable financial burden in low and middle-income countries, especially China [4, 5]. The Fifth Chinese National Health Services Survey (NHSS) in 2013 showed that 400 million Chinese people were diagnosed with at least one chronic disease. The 2014 WHO Global Status report on NCDs showed that they are now the most important causes of mortality worldwide, and cause about 38 million lives lost in 2012. By 2013, 85% of all deaths were due to chronic diseases, which caused about 70% of the total financial disease burden [6].

Chronic diseases impose heavy economic burdens on individuals, families and society. Family members with chronic diseases had a higher chance of experiencing health payment-induced poverty than those without, which is consistent in both developed countries and developing countries. In addition to the disease burden, chronic diseases may also impose a substantial economic cost [7]. Most low- and middle-income countries have relatively low levels of public expenditure on health care, inadequate health insurance, and relatively low coverage of health service. The shortfall in public expenditures on health care is usually made up by out-of-pocket (OOP) and other private health expenditures. So the effect of impoverishment caused by chronic diseases was rather heavily in the low- and middle-income countries [8]. According to a study conducted by Chunhong Jiang [9] showed that due to health expenses, an additional 10.53% of the families with a chronic patient got into poverty, which was more than twice the increase in the proportion of the families without a chronic patient.

The direct and indirect financial costs of chronic diseases to individuals and societies were enormous, while this trend still continues to grow. Direct costs include hospital costs, medical expenses, prevention costs, care-related travel, nursing, and family support costs. Indirect costs come from lower productivity, sick leave, loss of productive workers as a result of early retirement. These costs are heavy and can lead to poverty [10].

Many studies have shown that families with members who have chronic diseases face higher economic risks than other families, especially in developing countries, such as China [11, 12]. In rural China, the situation is even worse, and the economic burden of chronic disease has risen to the point where out-of-pocket health payments cause many households to fall into poverty, and others who have already in poverty sink deeper [13].

This study aims to explore the impact of chronic diseases on the health payment-induced poverty in Shaanxi Province, China. The findings of this study will provide evidence on the health payment-induced poverty and relevant factors in households with chronic disease. Our findings may contribute to improving and adjusting related health policy, thereby further alleviating the economic burden of chronic disease.

## Methods

### Data

The data was obtained from the Fifth National Health Service Surveys (NHSS) of Shaanxi Province, which was conducted in 2013. NHSS was organized and directed by the National Health and Family Planning Commission of China every 5 years. Shaanxi province is located in the northwest of China, covering an areas of over 205,800 Km^2^. In 2013, it had a population of 37.6 million and 48.7% of residents lived in rural areas.

A four-stage, stratified, random sampling method was used in 2013 to ensure the representation of the whole population. In total, 32 counties (districts) were randomly selected in Shaanxi Province in the first stage, 160 towns were sampled in the second stage. In the third stage, 320 villages (communities) were selected in sampled townships. In total, 20,700 households were identified.

The study used a survey and qualitative methods to collect and analysis data. During the survey, all households members were interviewed individually using a structured household questionnaire. Data from the household survey was used to analysis the effect of the percentage of chronic disease in households on the health payment-induced poverty. Questions related to this paper included: demographic and socio-economic information on individuals and households, self-reported illness and injury, reported outpatient and inpatient health service utilization, questionnaire to measure annual household expenditure on food, accommodation, transportation, and out-of-pocket health expenditure and so on. The recall period for household expenditure questions was 1 year prior to the survey. An adult who was fully aware of the household income and expenditure information was eligible to respond in this section. Although the data were self-reported, the literature suggested that self-reported income tended to under-true income in the survey [14]. So, following the literature, we used the household consumption expenditure to proxy economic status in this study.

Considering the fact that households living in rural areas are more likely to encounter poverty than households living in urban areas [3], we analysis the rural sample and urban sample separately.

### Health payment-induced poverty

Health payment-induced poverty refers to the poverty absolutely attributable to health payment, which is measured by the difference between poverty before health payments are subtracted from total household income and poverty after they are subtracted. It occurs when health payments in a given year actually push a household below the poverty line (health payment-induced poverty), or further below the poverty line. Household may adopt many changes to cope with health payment-induce poverty, which may influence household income and expenditure. The measurement of health payment-induced poverty assumes that household expenditure is not responsive to the health payment and variation of expenditure is ignored.

We use three indices to capture the effect of chronic disease on poverty incidence, poverty depth and poverty intensity in households: poverty headcount ratio, poverty gap and mean positive poverty gap [15].

Poverty headcount is measured as the households who fall below the poverty line due to out-of-pocket health payment as a proportion of all the households in the population. Let *x*_*i*_ be the per capita total expenditure of household *i*. An estimate of the gross of health payment poverty headcount is Eq. (1):

1 $$ H=\frac{\sum_{i=1}^N{s}_i{p}_i}{\sum_{i=1}^N{s}_i} $$

Where *p*_*i*_ = 1 if *x*_*i*_ < PL and is 0 otherwise, *s*_*i*_ is the size of the household, and *N* is the number of households in the sample.

Poverty gap (G) measures the percentage deficit from the poverty line of those households that have become poor due to out-of-pocket health payment. It is the “depth” of the poverty. The individual-level poverty gap is *g*_*i*_ = *p*_*i*_(*PL*-*x*_*i*_), then the poverty gap of the whole sample could be defined as Eq. (2):

2 $$ G=\frac{\sum_{i=1}^N{s}_i{g}_i}{\sum_{\mathrm{i}=1}^{\mathrm{N}}{s}_i} $$

The intensity of poverty is measured using the mean poverty gap (MPG), which represents the average deficit of the poor from the poverty line. Using Eq. (3):

3 $$ MPG=\raisebox{1ex}{$G$}\!\left/ \!\raisebox{-1ex}{$H$}\right. $$

To capture the effect of the percentage of chronic disease in the households on health payment-induce poverty, we firstly analysis the poverty measures before and after health payment in rural and urban areas respectively, then we analysis the influence of the percentage of chronic disease on the poverty before in rural and urban areas using logistic regression.

### Poverty line

To compute poverty counts and gap, a poverty line needs to be established. Poverty line are either absolute or relative. An absolute poverty line defines poverty in relation to an absolute amount of household expenditure per capita. An extreme absolute poverty line indicates the cost of reaching subsistence nutritional requirements only. A relative poverty line is defined as some fraction of mean or median household expenditure. In this study, to make the results comparable internationally, we adopted the international poverty line, US$ 1.25/day [16]. 2013 exchange rate: US $1 = 6.2 Yuan.

### Quality control

In order to ensure of the results of the survey to be accurate and reliable, the survey supervisors revisited 5% of the sampled households to check the accuracy of data recorded by interviews. In this process, 14 key questions were asked again to check the consistency of the information recorded. The consistency rates of the key questions recorded between the first and second visits was over 95%. The Mayer’s Index of this sample is 1.63, indicating that there was a superior representation of the whole population [17].

### Ethical considerations

This study was approved by the Ethics Committee of Xi’an Jiaotong University Health Science Center (approval number: 2015–644), and it conformed to the ethics guidelines of the Declaration of Helsinki.

### Statistical modeling

We modeled associations between selected socioeconomic characteristics and the odds of becoming poor due to health payment using logistic regression. Demographic characteristics including four variables: having children in the household, household size, household head’s gender, education of household head, household head’s marriage. Having children in the household is a dummy variable indicating whether there were members in the household below 5 years old. Health insurance characteristics including two variables: social health insurance (divided into four groups) and commercial health insurance (i.e. whether the household member have commercial health insurance or not). The percentage of chronic disease in the household were divided into four groups (the first group is that the percentage of chronic disease in the households was 0, the second group is 1~ 40, the third group is 41~ 50, the fourth group is 51~ 100). The utilization of health services including two variables: inpatient service usage (i.e. whether household members used inpatient services in the past 2 weeks) and outpatient usage (i.e. whether household members used inpatient services in the past year). The economic status of the household grouped into five quintiles. All of the analyses were performed using Stata 12.0. A two-sided *P* < 0.05 was established as the level of statistical significance for all tests.

## Results

### Characteristics of households

Characteristics of all households enrolled in the study were shown in Table 1. In total, 21,700 households were evaluated. In rural areas, 74.91% of household heads were male, and 25.09% were female. While in urban areas, 65.31% of households were male, 34.69% were female. The proportion of households with child under 5 years old were 13.13% in rural areas and 15.87% in urban areas. The percentage of householders with a junior high school or higher degree were 81.31% in rural areas and 90.11% in urban areas. The proportion of households with all members covered by social health insurance were 81.31% in rural areas and 90.11% in urban areas. The percentage of chronic disease in households was divided into four parts. The proportion of households having at least 1 member with self-reported chronic diseases in urban areas (42.85%) was significantly higher than that in rural areas (40.88%). Detailed characteristics of households were in Table 1.

**Table 1:** Characteristics of households enrolled in this study (%)

Variables	Description	Rural (*n* = 13,200)	Urban (*n* = 7500)
Household head’s gender	1^a^ if the head of household was male	74.91	65.31
	2 if the head of household was female.	25.09	34.69
Household size	1^a^ if 1–2 household members	32.13	44.48
	2 if 3–4 household members	46.71	44.89
	3 if more than 5 household members.	21.16	10.63
Having children in the household	1 if household having members below 5 years old	13.13	15.87
	0^a^ otherwise.	86.87	84.13
Household head’s educational level	1^a^ if the head of household was illiteracy,	18.69	9.89
	2 if the head of household was elementary,	31.58	17.21
	3 if the head of household was middle school,	35.81	32.52
	4 if the head of household was high school,	7.57	12.73
	5 if the head of household was university.	6.35	27.66
Household head’s marriage	1^a^ if the head of household was not married,	5.46	2.83
	2 if the head of household was married,	72.02	74.84
	3 if the head of household was else marriage	22.52	22.33
Outpatient service usage	1^a^ if household members used outpatient services in the past two weeks,	19.23	23.13
	2 if no household members used outpatient services in the past two weeks.	80.77	76.87
Inpatient service usage	1^a^ if household members used inpatient services in the past year,	9.11	10.50
	2 if no household members used inpatient services in the past year,	90.89	89.50
Social health insurance	1^a^ if having household members covered by UEMS,	1.04	25.38
	2 if having household members covered by URMS,	1.03	13.45
	3 if having household members covered by NCMS.	86.80	57.26
	4 else.	11.13	3.92
Commercial health insurance	1 if having household members covered by commercial health insurance,	4.81	5.22
	0^a^ otherwise.	95.19	94.78
Low-income family	1 if household was low-income family,	17.28	9.61
	0^a^ otherwise.	82.72	90.39
The percentage of chronic disease in the households	1^a^ if the percentage of chronic disease in the households was 0,	59.12	57.15
	2 if the percentage of chronic disease in the households was 1~ 40,	16.36	17.33
	3 if the percentage of chronic disease in the households was 41~ 50,	12.83	12.75
	4 if the percentage of chronic disease in the households was 51~ 100.	11.70	12.77
Economic status	1^a^ if the 20% low income households,	27.09	20.09
	2 if the 20% low middle income households,	21.14	19.91
	3 if the 20% middle income households,	18.05	21.33
	4 if the 20% high middle income households,	17.11	18.75
	5 if the 20% high income households.	16.61	19.92
Region	1^a^ if household in Shannan,	36.36	27.97
	2 if household was in Guanzhong,	45.45	56.03
	3 if household in Shanbei.	18.18	16.00

^a^reference groups

### Health payment-induced poverty in rural areas

Table 2 shows the poverty measures before and after health payment in different groups in rural areas. We defined that the percentage of chronic patients in the households was 0% as group 1, 1~ 40% as group 2, 41~ 50% as group 3 and 51~ 100% as group 4. Before health payment, 25.83% of households in group 1 were estimated to be in poverty, according to the international poverty line US $1.25/day. After health payment, the poverty headcount increases to 34.07%. A rise of 31.09% increase in poverty incidences. The estimated poverty gap rose 50.56%, from 832.77 Yuan pre-health payment to 1253.85 Yuan post payment. The mean positive poverty gap also increase from 3223.68 Yuan to 3679.98 Yuan, representing a deepening of the poverty of the already poor.

**Table 2:** Measures of poverty gross and net of health payments in different groups in rural areas

	Gross of health payment(1)	Net of health payment(2)	Difference	
			Absolute(3) = (2)–(1)	Relative(4) = [(3)/(1)]^a^100
Group 1^a^				
Poverty headcount^b^	25.83%	34.07%	8.24%	31.90%
Poverty gap(Yuan)	832.77	1253.85	421.08	50.56%
Mean positive poverty gap(Yuan)	3223.68	3679.98	456.3	14.15%
Group 2				
Poverty headcount	20.15%	31.03%	10.88%	54.00%
Poverty gap(Yuan)	775.67	1371.24	595.57	76.78%
Mean positive poverty gap(Yuan)	3849.82	4418.68	568.86	14.78%
Group 3				
Poverty headcount	28.35%	44.42%	16.07%	56.68%
Poverty gap(Yuan)	907.40	1717.66	810.26	89.29%
Mean positive poverty gap(Yuan)	2865.07	3611.78	746.71	26.06%
Group 4				
Poverty headcount	31.67%	47.47%	15.80%	49.89%
Poverty gap(Yuan)	3851.58	5632.40	1780.82	46.24%
Mean positive poverty gap(Yuan)	6513.52	7785.52	1272	19.53%

^a^Group 1: percentage of chronic patients in the households was 0%; Group 2: percentage of chronic patients in the households was 1~ 40%; Group 3: percentage of chronic patients in the households was 41~ 50%; Group 4: percentage of chronic patients in the households was 51~ 100%

^b^International poverty line, US$1.25/day. 2013 exchange rata: US$1 = 6.2 Yuan

In group 2, before and after health payment, a 50.56% increase in the poverty headcount, a 76.78% increase in the poverty gap and a 14.78% increase in the mean poverty gap. In group 3, due to the health payment, a 56.68 and 89.29% increase of the poverty headcount and poverty gap, respectively. In group 4, the increase in poverty headcount was 15.80%, in poverty gap was 49.89%.

### Health payment-induced poverty in urban areas

Table 3 shows the poverty measures before and after health payment in different groups in urban areas. We can see the same trend in urban and rural areas. In group 1, before health payment, 12.55% households were estimated to be in poverty, after health payment, the poverty headcount rises to 16.87%. In other three groups, despite the same trend when compared to rural areas, but we can see the households in urban areas suffer a bit lower impoverishment than rural areas. For all measures the difference in the estimate of poverty based on the household expenditure gross and net of health payments is statistically significantly different from zero at 5% or less.

**Table 3:** Measures of poverty gross and net of health payments in different groups in urban areas

	Gross of health payment(1)	Net of health payment(2)	Difference	
			Absolute(3) = (2)–(1)	Relative(4) = [(3)/(1)]*100
Group 1*				
Poverty headcount	12.55%	16.87%	4.32%	34.42%
Poverty gap(Yuan)	435.16	661.63	226.47	52.04%
Mean positive poverty gap(Yuan)	3466.71	3922.21	455.5	13.14%
Group 2				
Poverty headcount	9.38%	16.23%	6.85%	73.03%
Poverty gap(Yuan)	355.66	689.67	334.01	93.91%
Mean positive poverty gap(Yuan)	3789.79	4249.15	459.36	12.12%
Group 3				
Poverty headcount	11.19%	19.77%	8.58%	76.68%
Poverty gap(Yuan)	362.87	733.18	370.31	102.05%
Mean positive poverty gap(Yuan)	3242.07	3708.59	466.52	14.39%
Group 4				
Poverty headcount	14.61%	26.41%	11.80%	80.77%
Poverty gap(Yuan)	420.07	913.10	493.03	117.37%
Mean positive poverty gap(Yuan)	2874.47	3457.51	583.04	20.28%

*Group 1: percentage of chronic patients in the households was 0%; Group 2: percentage of chronic patients in the households was 1~40%; Group 3: percentage of chronic patients in the households was 41~50%; Group 4: percentage of chronic patients in the households was 51~100%

### Factors associated with health payment-induced poverty headcount

Table 4 provides the results obtained from the logistic regression models of health payment-induced poverty with other associated factors. As expected, most variables increased the risk of impoverishment. Interaction results show that, the percentage of chronic diseases in the households with health payment are 1.01 times to incur impoverishment in both rural areas and urban areas. With the member in the household increased, the chances of becoming impoverished increased. Inpatient health service usage increased the risk of suffering poverty 1.72 times in rural areas, and 1.62 times in urban areas. Compared to illiteracy, the household’s head who have the higher education level were less likely to be impoverished due to health payment both in rural areas and urban areas. The low-income family in rural areas were 2.06 times suffering health payment-induced poverty, whereas there was no significant in urban areas. The male household’s head was seen as the protective factor, the odds ratio (OR) was 0.55 (95%CI: 0.47–0.65) in rural areas and 0.68 (0.54–0.86) in urban areas. There was no statistically significant association between having children below 5 years old incurring poverty both in rural areas and urban areas.

**Table 4:** Determinants of poverty incidence in rural and urban areas of Shaanxi

	Poverty incidence	
	Rural areas OR(95%CI)	Urban areas OR(95%CI)
Health payment		
Yes	2.89(2.47–3.39) *	2.20(1.71–2.83) *
No	Ref	
The percentage of chronic disease in the households	0.99(0.99–1.00)	0.99(0.99–1.00)
Health payment × The percentage of chronic disease in the households	1.01(1.01–1.02) *	1.01(1.00–1.01) *
Household size		
1~ 2	ref	
3~ 4	3.72(3.10–4.46) *	1.65(1.21–2.25) *
≥ 5	13.99(9.88–19.80) *	4.19(2.31–7.62)*
Having children in the household		
Yes	1.28(0.20–7.78)	1.05(0.68–1.63)
No	Ref	
Household head’s gender		
Female	Ref	
Male	0.55(0.47–0.65) *	0.68(0.54–0.86) *
Household head’s educational level		
Illiteracy	Ref	
Elementary	0.55(0.46–0.67) *	0.83(0.62–1.01)
Middle school	0.40(0.33–0.49) *	0.56(0.41–0.75) *
High school	0.39(0.28–0.52) *	0.57(0.38–0.86) *
University	0.17(0.09–0.33) *	0.26(0.13–0.53) *
Household head’s marriage		
Unmarried	Ref	
Married	5.94(0.51–7.83)	3.32(0.82–6.01)
Else	2.54(0.92–3.39)	2.63(0.43–4.86)
Outpatient service usage		
Yes	0.97(0.81–1.15)	0.93(0.71–1.21)
No	Ref	
Inpatient service usage		
Yes	1.72(1.57–1.90) *	1.62(1.45–1.86) *
No	Ref	
Low-income family		
Yes	2.06(1.58–2.69) *	0.55(0.29–1.07)
No	Ref	
Region		
Guanzhong	1.36(1.17–1.58) *	1.26(0.99–1.60)
Shanbei	1.16(0.97–1.40)	0.55(0.39–0.41) *
Shannan	Ref	

*P ≤ 0.05

## Discussion

This study used the cross-sectional data from the 5th National Health Services Survey in Shaanxi Province to assess the effect of the percentage of chronic diseases in households on health payment-induced poverty. We measured the incidence, depth, and intensity of impoverishment health payment and provide strong evidence that medical expenditure is a financial burden for the households with chronic diseases members. We find that with the percentage of chronic diseases in the households growing, the health payment-induced poverty increases. Furthermore, rural households face more severe poverty problems than the urban areas residents. Male household head and the household head with higher educational level were regarded as the significant protection factors from impoverishment, and the family in urban Shanbei was also less likely to experience poverty.

From the overall perspective of China, with the rapid economic growth, the poor population have been greatly reduced. But there still a population of 43.32 million was in severe poverty in China in 2016, impoverishment from medical expenses are still persistent. Chronic diseases not only have serious negative effects on quality of daily life of patients, but also bring huge losses of welfare to their family. In our study, more than 40% of the households had at least one member with chronic disease. Our results show that those households are at higher risk to fall into poverty than these without a member with chronic disease.

According to our findings, the male household head was seen as a protective factor, in rural areas 0.55 and in urban areas the OR was 0.68, which is consistent with the former study [18]. We can explain this with the fact that male heads of households are always with a better conditions to provide the families with better economic status, such as the families main workforce, and the provider of the primary source of income of the family, so these households are expected to be healthy. Similar to the reports from other studies, household head’s educational level was found to be another important protective factor of health payment-induced poverty. The reason for this result can be explained as the educational level of the household head is related to the communication attitudes towards health and health-related behaviors in the family. Therefore, the household head with higher education level can protect the families’ health and reaction to health problems [19]. Unlike other studies [20, 21], our study found that the large household size was the risk factor of health payment-induced poverty. One of the potential reason is that the household with large size may have complicated family structure. The family with large size, maybe will have more patients or elders.

As expected, low-income households in rural areas are more likely to be poor due to health payment, while our research found that there was no statistically significant correlation with health payment-induced poverty in urban areas. This is mainly due to the limited ability of low-income households in rural areas to pay for non-living expenses, which is more likely to lead to poverty. In addition, low-income families are near the poverty line, which makes them more easier to fall into poverty or fall into deeper poverty, so in rural areas, many poor households will choose not to seek care when they get ill, rather than become impoverished [22]. Further efforts are needed to alleviate the financial burden of certain disadvantaged groups, such as the households with low-income.

In contrast to our expectation, we found that there was no significant association between impoverishment and households with a child or children, which was inconsistent with the study in Turkey [23]. Further studies are needed to explore the reason. Having inpatient patients in the household had a higher risk of incurring impoverishment.

Perhaps the main policy implication of our study is that policy makers need to understand whether there are any characteristics that make people more vulnerable to poverty caused by health payment, and should provide health insurance for the poor in order to reduce their vulnerability to health and financial shocks. Chronic diseases prevention is an important area for health policy development to reduce the economic burden of chronic diseases. Additionally, rural areas need a lot of investment because more than 70% of the poverty-stricken people live in the rural areas and they are the one who are more likely to fall into poverty.

Several limitations of our study should be acknowledged. Firstly, as the household income and consumer expenditures were both self-reported, recall bias might exist in the data, However, self-reported health service utilization and expenditure is widely used in the literature in the large-scale household survey. Secondly, the use of self-reported measures of chronic diseases may underestimate the prevalence of low- and middle-income households, particularly those with lower socioeconomic status. In addition, the chronic diseases were based on self-reported rather than being diagnosed by physicians, thus we could underestimate the disease burden. Thirdly, we used a higher poverty line than earlier studies, which use the poverty line of a dollar per day [5]. When compared with these studies, the poverty headcount, poverty gap and mean positive poverty gap may be overestimated. However, we centered around the relative differences of the poverty indexes before and after health payment, so the conclusion of our study would be reliable no matter which poverty line was used.

## Conclusions

With the percentage of chronic patients in the households growing, the health payment-induced poverty increases. Furthermore, the households with more chronic disease in rural areas were more likely to suffer poverty than those in urban areas. Our analysis emphasizes on the need to protect households from the impoverishment of chronic diseases.
